# Evidence That Descending Cortical Axons Are Essential for Thalamocortical Axons to Cross the Pallial-Subpallial Boundary in the Embryonic Forebrain

**DOI:** 10.1371/journal.pone.0033105

**Published:** 2012-03-08

**Authors:** Yijing Chen, Dario Magnani, Thomas Theil, Thomas Pratt, David J. Price

**Affiliations:** Centre for Integrative Physiology, University of Edinburgh, Edinburgh, United Kingdom; Tokyo Medical and Dental University, Japan

## Abstract

Developing thalamocortical axons traverse the subpallium to reach the cortex located in the pallium. We tested the hypothesis that descending corticofugal axons are important for guiding thalamocortical axons across the pallial-subpallial boundary, using conditional mutagenesis to assess the effects of blocking corticofugal axonal development without disrupting thalamus, subpallium or the pallial-subpallial boundary. We found that thalamic axons still traversed the subpallium in topographic order but did not cross the pallial-subpallial boundary. Co-culture experiments indicated that the inability of thalamic axons to cross the boundary was not explained by mutant cortex developing a long-range chemorepulsive action on thalamic axons. On the contrary, cortex from conditional mutants retained its thalamic axonal growth-promoting activity and continued to express *Nrg-1*, which is responsible for this stimulatory effect. When mutant cortex was replaced with control cortex, corticofugal efferents were restored and thalamic axons from conditional mutants associated with them and crossed the pallial-subpallial boundary. Our study provides the most compelling evidence to date that cortical efferents are required to guide thalamocortical axons across the pallial-subpallial boundary, which is otherwise hostile to thalamic axons. These results support the hypothesis that thalamic axons grow from subpallium to cortex guided by cortical efferents, with stimulation from diffusible cortical growth-promoting factors.

## Introduction

The precise and often highly complex connectivity of nervous systems develops through the guidance of elongating axons along specific pathways at specific times. As nervous systems grow in complexity, they contain an ever-increasing number of features that might provide guidance cues to growing axons. For example, cells or axons made at one stage in development might be used to guide axons generated at a later stage. The evidence that this occurs came initially from work on invertebrates in which guiding cells (called guidepost cells) were selectively destroyed, causing defects of later-growing axons [1]. It has been hypothesized that this also occurs in highly complex neural structures such as the mammalian forebrain [2], [3], [4], [5], [6], but supporting evidence has been hard to obtain, due largely to the technical difficulty of selectively destroying potential guidance cells in relatively inaccessible embryos.

The cerebral cortex receives sensory afferents from the thalamus via thalamocortical axons. During development, axons from the thalamus are thought to be guided towards the cortex by a variety of mechanisms, many of which remain poorly defined [6], [7]. The strongest hypotheses for which there is both *in vitro* and *in vivo* evidence focus on the important guiding role of intermediate cells in the subpallium. These cells form a permissive corridor through which thalamic axons grow towards the pallium and they express gradients of guidance molecules such as Netrin-1 and Ephrin-A5 that maintain the topographic order of thalamic axons as they traverse this region [8], [9], [10], [11], [12]. How thalamocortical axons are guided beyond the subpallium and into the pallium remains unclear. Whereas the pallium does exert a growth-promoting effect on thalamic axons, there has been no clear demonstration that it releases a chemoattractant drawing thalamic axons towards it [3], [10], [13]. Attention has focussed, therefore, on the descending axons of cortical neurons as a potential source of guidance for ascending thalamocortical axons navigating towards the cortex [2], [4], [14], [15], [16], but as yet there is no strong supporting *in vivo* evidence for this hypothesis.

Unlike the situation in invertebrates, controlled deletion of selected cells such as those with descending cortical efferents is difficult to achieve in the embryonic mammalian brain. Although it is possible to generate transgenic mice in which specific cells are made to produce cellular toxins, thereby eliminating those cells [17], this approach is liable to cause large morphological distortions that themselves might interfere with axonal growth. Here, we used a conditional transgenic method to block the maturation of cerebral cortical neurons by the cortex-specific deletion of an intracellular protein, adenomatous polyposis coli (APC). APC is a large multifunctional tumour-suppressor protein [18] important for several processes including cytoskeletal dynamics, cell polarity and neurite outgrowth [19], [20], [21]. In our conditional null mutants, a cortical progenitor layer develops but it does not generate the postmitotic neurons that would project axons subcortically. This allowed us to analyse the extent to which the absence of descending cortical axons affects the development of ascending thalamocortical axons.

We found that blocking cortical efferent development did not stop thalamic axons traversing the subpallium in an ordered way but did completely prevent them from crossing the pallial-subpallial boundary (PSPB) to enter the cortex. Instead, thalamic axons turned away from the cortex some distance before the PSPB and before they encountered any mutant tissue. We carried out experiments to show that this could not be explained by the null mutant cortex developing long-range chemorepulsive properties, nor by its losing its normal growth-promoting effect on thalamic axons. These results suggested that the failure of thalamic axons to enter the null mutant cortex was due to the loss of descending cortical efferents. In further support of this we found that thalamic axons from conditional null mutants could be induced to cross the PSPB into the cortex if wild-type cortex was substituted for mutant cortex, thereby reintroducing descending corticofugal axons. These results provide the clearest evidence to date for the importance of cortical efferents in guiding thalamocortical afferents across the PSPB and into the pallium.

## Materials and Methods

### Ethics Statement

All mice were bred in-house in line with Home Office, UK, legislation. The licence authorising this work was approved by the University of Edinburgh's Ethical Review Committee on 22nd September 2008 (application number PL35-08) and by the Home Office on 6th November 2008. Animal husbandry was in accordance with the UK Animals (Scientific Procedures) Act 1986 regulations. The *Apc^580S^* floxed allele (Shibata et al, 1997) was conditionally inactivated using *Emx1^Cre^* [22], which is expressed in the cerebral cortex from E9.5. Embryos in which both copies of *Apc* were deleted (*Emx1^Cre/+^Apc^580S/580S^*) or in which only one or neither copy of Apc was deleted (controls: *Emx1^Cre/+^Apc^580S/+^*, *Emx1^+/+^Apc^580S/580S^* or *Emx1^+/+^Apc^580S/+^*) were genotyped as described before [23]. The activity of Cre recombinase was confirmed using a floxed-stop-*LacZ* reporter allele [24]. In addition, mice carrying ubiquitously expressed tau-GFP were used for culture experiments [25]. The first day of vaginal plug was designated as E0.5. Pregnant females were killed by cervical dislocation and embryos were removed immediately. *Emx1^Cre^*; *Apc^loxP/loxP^* mice died at birth, possibly because defects of their mouths prevented them suckling normally.

### Immunohistochemistry

Embryos were fixed in 4% paraformaldehyde in phosphate buffered saline (PBS) for 2 to 4 hours (cryostat sectioning) or overnight (paraffin or vibratome sectioning) at 4°C on a rocking platform. Embryonic heads were either: embedded in wax and cut into paraffin sections 10 µm thick; frozen and cut into cryostat sections 20 µm thick; or embedded in agarose and cut into vibratome sections 100 µm thick. Immunohistochemistry was performed on coronal sections. The following primary antibodies were used: anti-Apc (ab15270), rabbit, 1∶100 (Abcam); anti-Pax6 (PAX6), mouse, 1∶200 (Developmental Studies Hybridoma Bank [DSHB]); anti-L1 (MAB5272), rat, 1∶500 (Chemicon International); anti-Tag1 (4D7/TAG1), mouse, 1∶100 (DSHB); anti-Gsh2, mouse, 1∶1500 [26]; anti-microtubule associated protein (anti-MAP2) (AB5622), mouse, 1∶500 (Upstate Cell Signalling Solutions); anti-neurofilament (NA1297), mouse, 1∶500 (Biomol International, L.P.); anti-RC2 (RC2), mouse, 1∶100 (DSHB). Chromogenic visualization was done with the Envision^+^ Kit (Dako). For immunofluorescence, following incubation with the primary antibody, sections were incubated with species specific secondary antibodies conjugated to Alexafluor-488 or Alexafluor-568 (Molecular Probes). Nuclei were counterstained using the DNA dye TO-PRO-3 iodide (Molecular Probes) at 1∶100 dilution in water.

### *In Situ* Hybridization

Brains of mouse embryos were fixed overnight in 4% paraformaldehyde in PBS before processing to wax blocks. Wax sections were cut at 10 µm and RNA probes were labelled with digoxigenin. *In situ* hybridizations for *Ig-Nrg1* were performed as previously described [27].

### Tract-tracing

To label specific axon tracts, brains fixed in cold 4% paraformaldehyde in PBS were injected at discrete points with DiI and DiA lipophilic axonal tracers (Molecular Probes). For each embryo, the brain was separated into two halves by cutting along the midline. Tips of glass micropipettes coated with DiI or DiA crystals were used to impale the embryonic neocortex after the pia was removed. For each half of the brain, DiI and DiA crystals were placed in the thalamus, with DiI at the anterior part and DiA at the posterior part of the thalamus. After 2–4 weeks at room temperature to allow the diffusion of the fluorescent dyes along the axons, vibratome sections were cut coronally at 100 µm, counterstained with TO-PRO 3 and examined using a confocal microscope.

### Explant cultures

E14.5 embryos were collected in Earle's balanced salt solution. Thalamic explants (500×500×250 µm) were taken from tau-GFP^+^ embryos so that thalamic axons could be distinguished from tau-GFP^−^ cortical axons using anti-GFP antibodies. Co-cultured pallial and subpallial tissues were from experimental (*Emx1^Cre/+^Apc^580S/580S^*) or control (*Emx1^Cre/+^Apc^580S/+^*, *Emx1^+/+^Apc^580S/580S^* or *Emx1^+/+^Apc^580S/+^*) embryos. Collagen (10–20 µl) was spread evenly in the wells of a 4-well culture dish and allowed to set. In each well, one thalamic explant was placed on top of the collagen either alone or adjacent to one explant from the pallium or from the hypothalamus. Another 20 µl of collagen was added to submerge the explants and allowed to set; 500 µl of serum free culture medium was then added into each well and the explants were incubated at 37°C for 3 days. Cultures were fixed in 4% paraformaldehyde before being immunoreacted to reveal GFP (rabbit anti-GFP, 1∶3000, Abcam; secondary antibody: goat anti-rabbit Alexa Fluor 568, 1∶200, Molecular Probes). Cultures were counterstained with TO-PRO-3 (1∶1000). Images of each culture were acquired by confocal microscopy. Quantitative data on the amount and direction of outgrowth from each thalamic explant was obtained with the Image J programme, used as described in the context of our results [28]. Statistics used to compare outgrowth in different co-culture paradigms included ANOVA, Tukey tests and Student's t-tests.

### Organotypic slice cultures

E14.5 embryos were collected in ice cold Krebs solution and brains were removed and embedded in low melting point agarose. Coronal sections (300 µm) were cut on a vibratome and placed on polycarbonate membranes in Eagle's minimum essential medium, incubated for 1 h (37°C, 5% CO_2_), and transferred to Neurobasal medium. Transplants were cut with fine scissors from the donor slice and transferred to the host slice with fine forceps. The slices were incubated at 37°C, 5% CO_2_ for 3 days to allow axons to grow. Slices were fixed in cold 4% paraformaldehyde in PBS overnight. Red lipophilic axonal tracers were injected into thalamic transplants; green lipophilic axonal tracers were injected into the cortex. The slices were kept for 2 weeks at room temperature to allow the diffusion of the fluorescent dyes along the axons, were transferred to 1∶1 Glycerol/PBS, were counterstained with TO-PRO 3 and were examined using a confocal microscope.

## Results

### Effects of cortex-specific deletion of Apc on neuronal generation and efferent development

**Figure 1:**
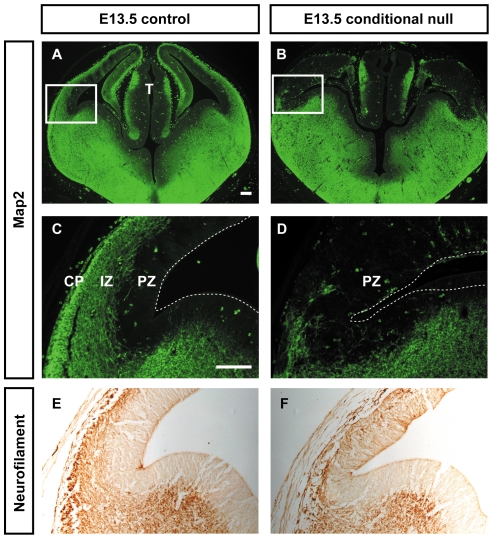
Cortex-specific deletion of *Apc* disrupts the generation of differentiating neurons. (**A–D**) Map2 immunohistochemistry on coronal sections of E13.5 control and conditional null mutant forebrain. Areas boxed in **A** and **B** are shown at higher magnification in **C** and **D**, respectively. (**A,C**) in the pallium of controls, Map2 was detected through the cortical plate (CP) and the intermediate zone (IZ), both of which are thicker laterally; (**B,D**) in contrast, there were relatively few Map2-positive cells in the pallium of conditional null mutants (PZ, proliferative zone; T, thalamus). (**E,F**) Neurofilament (NF) immunohistochemistry on coronal sections of E13.5 control and conditional mutant forebrain: (**E**) a superficial layer of immunostaining was detected in the pallium of control brains but (**F**) not of conditional mutant brains. Scale bars: **A&B**, 100 µm; **C–F**, 100 µm.

We studied the effects of cortex-specific loss of APC in *Emx1^Cre^*; *Apc^loxP/loxP^* embryos (referred to here as conditional null mutants) using Map2 immunohistochemistry to mark post-mitotic neurons [29], [30]. In the lateral cortex of controls, Map2 was detected through the depth of the differentiating layers superficial to the progenitor zone with strongest staining in the developing cortical plate and intermediate zone (Fig. 1A,C). In E13.5 conditional null mutants, there were relatively few Map2 positive cells throughout the entire cortex (Fig. 1B,D). This was associated with a corresponding loss of immunoreactivity for the axonal marker neurofilament (Fig. 1E,F). The expression patterns of Map2 and neurofilament in the subpallium and thalamus were similar in the conditional null mutants and in the controls (Fig. 1A,B,E,F), in agreement with our previous findings that subpallial and thalamic morphology and expression of marker genes such as Islet1, Olig2, Gli1 and Ptc were unaffected in these regions in these mutants [23] (see also Results below). We conclude that conditional null mutants have a cortex-specific inability to generate significant numbers of differentiating neurons at E13.5.

**Figure 2:**
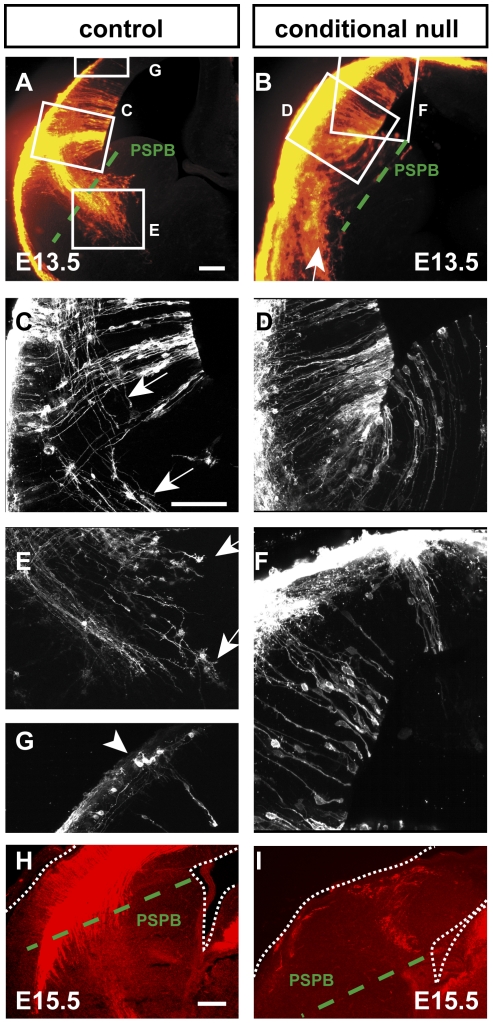
Cortex-specific deletion of *Apc* prevents the production of descending cortical efferents. Injections of axonal tracers into the cortex of control and conditional null mutant embryos: areas boxed in **A** are shown at higher magnification of single optical sections in **C,E,G** and areas boxed in **B** are shown at higher magnification in **D,F**. (**A,C,E,G**) In E13.5 controls, cortical efferents descended across the PSPB: arrows indicate axons and growth cones descending from labelled cortical cell bodies (arrowhead in **G**). (**B,D,F**) In E13.5 conditional mutants, there were no cortical efferents crossing the PSPB (arrow in **B** indicates a group of pallial cells that were labelled retrogradely from the injection site). (**H,I**) Tag1 immunohistochemistry on coronal sections of E15.5 control and conditional mutant forebrain: (**H**) large numbers of descending Tag1 positive cortical efferents were detected in the control brains but (**I**) not in the conditional mutant brains. Scale bars: **A&B**, 100 µm; **C–F**, 100 µm; **H&I**, 100 µm.

To test for descending cortical efferents, DiI crystals were injected into the lateral cortex at both rostral and caudal levels in E13.5 control and conditional null mutant embryos (Fig. 2A,B). In E13.5 control embryos, large bundles of cortical efferents were observed projecting from the cortex, crossing the PSPB and starting to traverse the subpallium (Fig. 2A). With confocal microscopy, individual labelled axons could be traced across the PSPB (e.g. arrows in Fig. 2C,E). We observed retrograde labelling of superficially located cortical neurons medial to the DiI injections (e.g. arrowhead in Fig. 2G). DiI was also picked up by radial glial processes (Fig. 2C). In contrast, there was no sign of cortical efferents descending across the PSPB in conditional mutants at E13.5 at any rostrocaudal level (Fig. 2B,D,F). As in controls, labelled radial processes were visible in the mutant ventricular zone (Fig. 2D,F). The absence of descending cortical efferents in mutants was confirmed by the loss of immunoreactivity for the cortical axonal marker Tag1 (Fig. 2H,I). Normally, large numbers of Tag1-positive cortical efferents descend across the PSPB from embryonic cortex (Fig. 2H) but none were observed in conditional null mutants (Fig. 2I).

**Figure 3:**
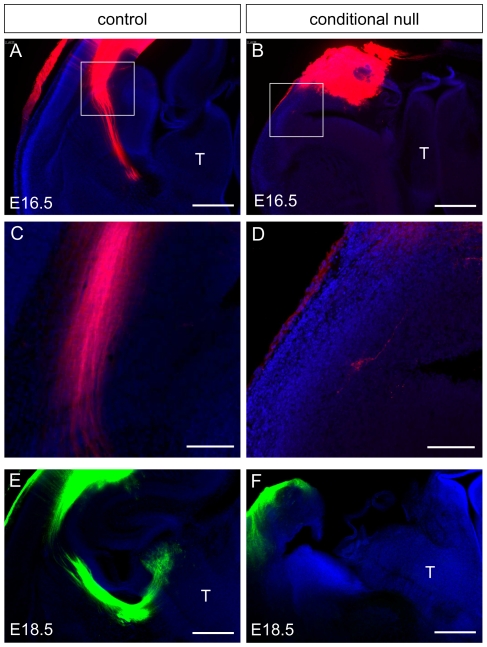
Corticothalamic and thalamocortical axons were not detected in E16.5–E18.5 conditional null mutants. (**A,B**) Placements of axonal tracers into the E16.5 cortex of control and conditional null mutant embryos: areas boxed in **A** and **B** are shown at higher magnification in **C** and **D**, respectively. (**A,C**) Cortical placements into control brains at E16.5 labelled large bundles of axons that were travelling through the ventral telencephalon. (**B, D**) Cortical placements into conditional null mutants at E16.5 failed to label more than the occasional axon navigating in the direction of the PSPB. (**E**) At E18.5, cortical placements labelled thalamic (T) nuclei in controls. (**F**) Cortical placements into conditional null mutants at E18.5 failed to produce any labelling of the thalamus. Nuclei were counterstained with TO-PRO-3 iodide. Scale bars: **A&B**, 500 µm; **C&D**, 100 µm; **E&F**, 500 µm.

Similar results were obtained in older conditional null mutants: cortical injections of DiI at E16.5 labelled only the occasional axon that appeared to be navigating ventrally in the direction of the PSPB (Fig. 3B,D), whereas in the control brains bundles of axons descending from the cortex were labelled (Fig. 3A,C). Cortical placements of DiA crystals at E18.5, which would normally anterogradely label corticothalamic axons as well as retrogradely label afferents originating from the thalamus (Fig. 3E), resulted in no thalamic label in mutants at all (Fig. 3F).

We conclude that, from the age when cortical connections would normally be first forming, cortex-specific deletion of *Apc* severely depletes the cortex of differentiating neurons and prevents the cortical production of descending efferents.

### Thalamocortical axons fail to cross the PSPB in conditional mutants

**Figure 4:**
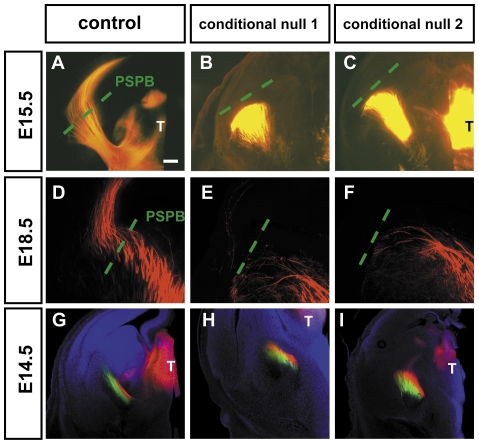
Thalamic axons failed to reach the cortex in *Apc* conditional null mutants. (**A,D**) At E15.5 and E18.5, injections of tracer in control thalamus labelled large bundles of axons projecting from the thalamus (T) through the subpallium and the intermediate zone of the cortex. (**B,C**) In E15.5 conditional null mutants, thick bundles of axons were observed traversing the subpallium but they stopped before crossing the PSPB and entering the cortex; instead, they changed direction and turned ventrally. (**E,F**) In E18.5 conditional null mutants, labelled thalamic axons deflected away from the PSPB and cortex. (**G–I**) In these E14.5 embryos, DiI (red) was injected in rostral thalamus whereas DiA (green) was injected in caudal thalamus (the DiA injection sites are not seen in these planes of section). Nuclei were counterstained with TO-PRO-3 iodide. (**G**) In controls, the thalamic axons from the rostral thalamus maintain their position medial to axons from the caudal thalamus as they traverse the subpallium. (**H,I**) Results from two conditional null mutants show that the same order is maintained up to the point at which the thalamic axons halt their progress toward the cortex. Scale bar: **A–I**, 100 µm.

In control embryos DiI crystals placed into the thalamus at E15.5 or E18.5 labelled large bundles of thalamocortical axons (Fig. 4A,D). In conditional null mutants (Fig. 4B,C,E,F), dense bundles of thalamic axons traversed the subpallium of conditional null mutants but they stopped and many axons turned ventrally, perpendicular to their normal trajectory, as they approached the PSPB. Out of three conditional null mutant E18.5 brains, a total of only three labelled axons were observed running through the cortex in one case, indicating an almost complete and persistent failure of any thalamic axons to enter the cortex.

To test whether the earlier growth of thalamic axons through the subpallium was topographically organised we injected DiI and DiA in the rostral and the caudal part of the thalamus, respectively. Thalamocortical axons from the rostral thalamus normally grow through the subpallium in a medial location to axons from the caudal thalamus. This pattern can be seen in Fig. 4G, where red (DiI) axons from rostral thalamus are medial to green (DiA) axons from caudal thalamus. This relative organisation of these two sets of axons is preserved in the thalamic axonal tracts of conditional null mutants (Fig. 4H,I). We conclude that cortical efferents are not required for this level of topographic organisation of developing thalamocortical tracts.

In E14.5 control embryos, the bundles of thalamic axons approaching the PSPB are narrow, suggesting tight fasciculation of their axons (Fig. 4G). The bundles of thalamic axons in the equivalent position in E14.5 conditional null mutants are broader, suggesting that they might be less tightly fasciculated (Fig. 4H,I). During normal development in mice, thalamic afferents and cortical efferents interact in the ventral telencephalon at around E14.5 and the absence of this interaction might explain why thalamic afferents are less tightly fasciculated as they approach the cortex. This phenomenon becomes more obvious in conditional null mutants at E15.5, where thalamic axons change their directions close to the PSPB and make a sharp turn ventrally towards the basal telencephalon (Fig. 4B,C).

**Figure 5:**
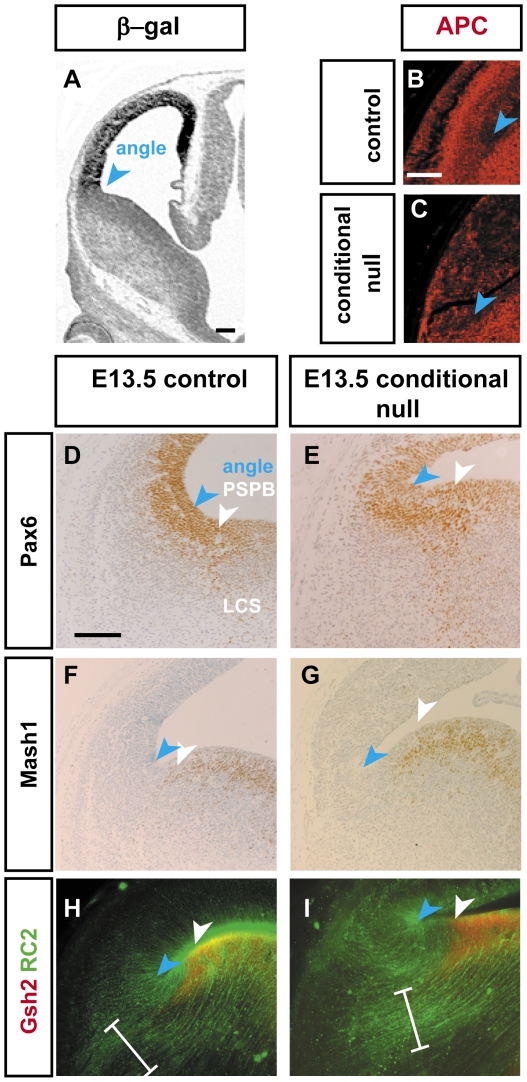
The expression of maker genes of PSPB is similar between control and conditional null mutant. (**A**) A *LacZ* reporter allele shows that *Emx1^Cre^* causes cortical recombination as far ventrally as the angle region at E13.5. (**B,C**) Expression of APC around the angle region (blue arrowhead) in (**B**) control and (**C**) conditional null mutants at E13.5, confirming loss of APC dorsal to the angle region, in the region labelled for β-galactosidase in **A**. (**D**) The E13.5 PSPB (white arrowhead), situated ventral to the angle region of the cortex (blue arrowhead), is marked by the transition from high (dorsal) to lower (ventral) expression of the transcription factor Pax6 and by cells of the lateral cortical stream (LCS). (**E**) In the conditional null mutant, Pax6 expression at the PSPB is similar to the control in **D**. (**F,G**) At E13.5, the expression pattern of the transcription factor Mash1 is similar between the control in **F** and the conditional null mutant in **G**. Expresses is in the subpallium and displays a sharp boundary respecting the medial edge of the PSPB (white arrowhead). (**H,I**) Similar to the control in **H**, the E13.5 PSPB in a conditional null mutant in **I** is marked by the lateral edge of the expression domain of ventrally-expressed transcription factor Gsh2 and a prominent RC2-expressing radial glial palisade from this region flanks the PSPB (bracket). Scale bars: **A**, 200 µm; **B&C**, 200 µm; **D–I**, 200 µm.

We next examined the nature of the PSPB in conditional null mutants so that we could relate the defective growth of thalamic axons to it in more detail. The deletion of APC did not extend as far ventrally as the PSPB; evidence for this is shown in Fig. 5. Figure 5A shows the expression of a *lacZ* reporter gene [24] resulting from Cre-induced deletion by the *Emx1^Cre^* allele: as anticipated from the known expression pattern of *Emx1* [31], reporter expression extends only as far ventrally as the telencephalic angle region. The telencephalic angle region is a morphologically very obvious sharp angle at the base of the cortex shown by blue arrowheads in Fig. 5. APC immunostaining in conditional null mutants confirmed a loss of APC from the pallium only as far ventrally as the angle region (Fig. 5B,C). The embryonic PSPB (white arrowheads in Fig. 5) is a line ventral to the angle. At the PSPB, there is an abrupt change in regulatory gene expression patterns from those of the pallium to those of the subpallium [32]. For example, the transcription factor Pax6 is expressed at high levels on the pallial side of the PSPB but at low levels on the subpallial side (Fig. 5D), whereas the transcription factors Mash1 and Gsh2 are expressed only on the subpallial side at E13.5 (Fig. 5F,H). In conditional null mutants, the strip of ventral pallial tissue about 200 µm wide between the angle region and the PSPB (between the arrowheads in Fig. 5D–I) would not have suffered *Apc* deletion and, indeed, appeared normal when stained for markers of the PSPB (Pax6, Mash1 and Gsh2 expression are shown in Fig. 5E,G,I).

**Figure 6:**
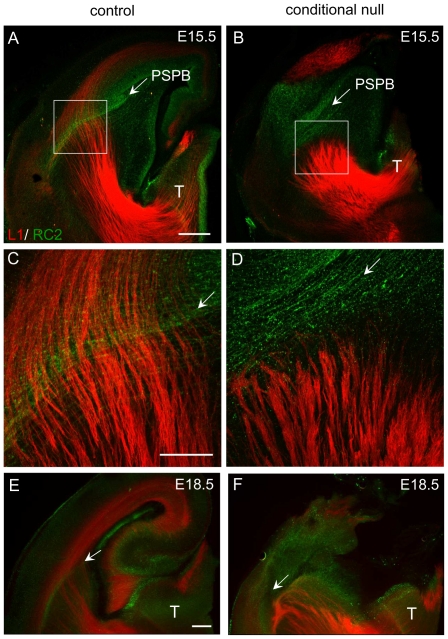
Thalamic axons stop before the radial glial palisade that flanks the PSPB. (**A,C,E**) In controls, L1 labelled thalamocortical axons pass the radial glial palisade and the PSPB to enter the cortex. (**B,D,F**) In conditional null mutants, L1 labelled thalamic axons stop at the lateral edge of the radial glial palisade. Boxed areas in **A** and **B** are shown at higher magnification in **C** and **D**. Scale bars: **A&B**, 200 µm; **C&D**, 200 µm; **E&F**, 200 µm.

From E13.5 onwards a characteristic palisade of RC2-expressing radial glial cells flanks the PSPB [33] (Fig. 5H, bracket). This was also the case in conditional null mutants (Fig. 5I). The medial part of the palisade (bracket) can be traced back to the Gsh2-positive subpallial ventricular zone, indicating that its medial edge is on the ventral side of the PSPB. This palisade is even more obvious at E15.5. A control is shown in Fig. 6A,C with thalamocortical axons crossing the palisade. A conditional null mutant is shown in Fig. 6B,D with thalamic axons stopping at the palisade's medial edge (Fig. 6D). Similarly, in E18.5 conditional null mutants, bundles of thalamic axons turned ventrally as they approached the medial side of the palisade (Fig. 6F, arrow), whereas in the control embryos thalamic axons crossed the palisade into the cerebral cortex (Fig. 6E, arrow). Taking all these data together indicates that the thalamic axons in conditional null mutants stop before they cross the palisade that flanks the PSPB and hence approximately 200 µm short of cortical *Apc*-deleted tissue: the effects of *Apc* deletion on thalamic axons are, therefore, at a distance from the site of deletion. Our evidence indicates that the PSPB and subpallium of the conditional null mutants are normal and so it is very unlikely that they contribute to the misrouting of thalamocortical axons as they approach the PSPB. Our findings are compatible with the possibility that the ascending thalamocortical axons do not cross the PSPB because descending cortical efferents are required to provide guidance across the border.

### Mutant cortex continues to promote thalamic axon growth

**Figure 7:**
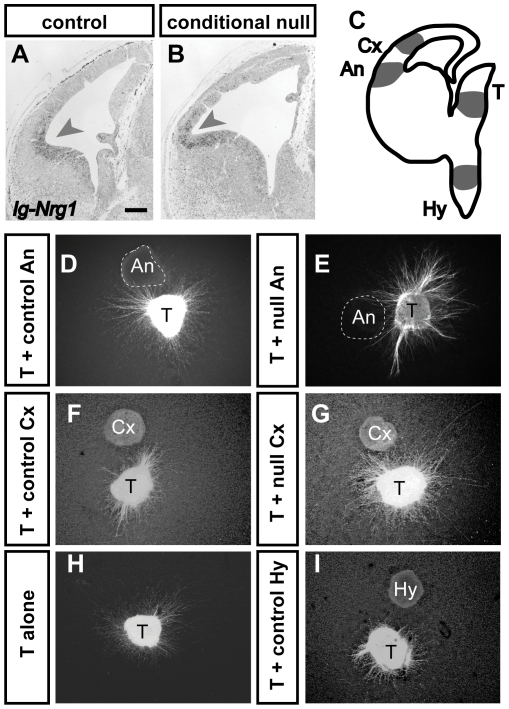
Control and *Apc^−/−^* angle region express molecules that stimulate thalamic growth. (**A, B**) *In situ* hybridizations show expression of *Ig-Nrg1* with highest intensity around the angle region (arrowheads). Scale bar: 200 µm. (**C**) This diagram shows the regions from which explants were taken for co-culture experiments: Cx, cortex; An, angle region; T, thalamus; Hy, hypothalamus. (**D–I**) Examples show six cultures, each containing thalamus and a different co-cultured tissue or, in **H**, thalamus alone.

The following experiments were designed to test an alternative possibility that a loss of growth-promoting molecules or the upregulation of inhibitory molecules diffusing from the conditional null mutant cortex might prevent, at a distance, the progress of thalamic axons across the PSPB. Evidence to date implicates the diffusible form of Neuregulin-1 (known as Ig-Nrg1), which is expressed in the E13.5 telencephalic ventricular zone with highest levels close to the angle region, as a required stimulant of thalamic axonal growth in this region [10]. The expression patterns of *Ig-Nrg1* were very similar in controls and conditional null mutants (Fig. 7A,B).

We then tested the ability of the cortex of control or conditional null mutant embryos to stimulate thalamic growth. Explants of E14.5 control or null mutant angle region or lateral cortex (labelled “An” and “Cx” respectively in Fig. 7C–G) were co-cultured in collagen gels about 200 µm away from E14.5 thalamic explants from control GFP-expressing embryos (labelled “T” in Fig. 7C–I). In addition, some thalamic explants were cultured alone and others were co-cultured with control hypothalamus (labelled “Hy” in Fig. 7C,I). Examples of cultures are shown in Fig. 7D–I. There were some trends from visual inspection. Firstly, there appeared to be more thalamic axonal outgrowth in the presence of angle region or lateral cortex (Fig. 7D–G) than when thalamic explants were cultured alone (Fig. 7H). Secondly, only hypothalamus appeared to direct outgrowth, preferentially in an opposite direction (Fig. 7I). To further investigate these trends, a statistical analysis of quantitative data was performed.

**Figure 8:**
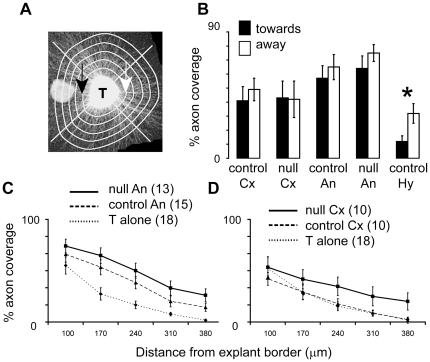
The cortex of conditional null mutants maintains the ability to promote thalamic axonal growth. Quantitative analyses show that neither the angle region nor more dorsal cortex of conditional null mutants have a reduced ability to promote thalamic axonal growth. (**A**) Concentric lines 100, 170, 240, 310 and 380 µm from the edge of the thalamic explant, divided into four quadrants, one facing towards the co-cultured explant, the other facing away. Within selected quadrants or along the entire length of each line, the percentages of the lengths that were crossed by axons were calculated. (**B**) Average (± sem) values for growth towards or away from co-cultured tissue, measured along the inner-most lines, as shown in A by black and white arrows. The only tissue that caused a significant difference in outgrowth on the two sides was hypothalamus (p<0.05; Student's t-test; n = 13), which is known to be repulsive to thalamic axons. (**C,D**) Average (± sem) values for growth across the entire length of each concentric line at each distance from each explant's edge (excluding parts covered by the co-cultured explant). (**C**) Results from thalamic explants cultured either alone or with null mutant or control angle regions (numbers of cultures in brackets). (**D**) Results from thalamic explants cultured either alone or with null mutant or control lateral cortical regions (numbers of cultures in brackets).

Outgrowth from all cultures was measured as shown in Fig. 8A and follows the method described in [34]. Briefly, a line was drawn around the circumference of each thalamic explant and each point on the line was then moved radially by 100, 170, 240, 310 and 380 µm to give a set of concentric polygons shown in Fig. 8A. Each polygon was cut into four segments by two orthogonal lines that crossed in the centre of each explant, thereby defining four quadrants, one of which faced towards the co-cultured explant (Fig. 8A). The proportion of each segment that was crossed by axons was calculated to give a value referred to here as the “% axon coverage”; axons were identified as having pixel intensities above the average background level, which was calculated from regions of the image that contained no cellular material.

We first compared the average % axon coverage of the inner-most concentric lines in quadrants facing either towards (black arrow in Fig. 8A) or away from (white arrow in Fig. 8A) a co-cultured explant: the results are in Fig. 8B. When thalamus was co-cultured with control or null mutant angle region or with control or null mutant lateral cortex, there were no significant differences between the values in the quadrants facing towards *versus* the values in the quadrants facing away from the co-cultured explant (first four sets of bars in Fig. 8B; numbers of cultures are 10–18, stated in brackets in C,D). When thalamus was co-cultured with hypothalamus, however, values for growth toward the hypothalamus were significantly lower than values for growth away from it (fifth set of bars in Fig. 8B; 5 cultures; Student's t-test, p<0.05); this is consistent with previous findings that the hypothalamus repels thalamic axons in such explant cultures and acts as a positive control for the ability of our culture technique to reveal repulsive interactions [35], [36].

We then carried out an analysis of the overall amount of outgrowth, irrespective of its direction, from thalamic explants cultured either alone or with control or null mutant explants from the angle region or from the lateral cortex. At each distance from each explant's edge, values were calculated for % axon coverage of the entire length of each concentric polygon (excluding the quadrant facing the co-cultured explant, within which some lines crossed the co-cultured explant preventing intersecting axons from being seen). Average values at each distance from the thalamus for all thalamic cultures and co-cultures are shown in Fig. 8C,D. This analysis gives a comprehensive view of the average amounts of outgrowth from the thalamus under each condition, taking into account both numbers and lengths of axons.

Two-way repeat measures ANOVA on all of the data combined indicated that there was a significant effect of co-cultured tissue and of its type (p<0.05). The greatest effect was stimulation of thalamic outgrowth by angle region, both control and conditional null (Fig. 8C). Explants of angle region stimulated more outgrowth than explants of lateral cortex (compare values in Fig. 8C and D; this tendency is also seen in Fig. 8B), in line with this region's higher expression of Ig-Nrg1 (Fig. 7A,B). Whereas the numbers of axons growing from thalamus cultured alone reached zero at the maximum distance measured, significant numbers of axons grew beyond this in co-cultures with angle region (control or conditional null; Fig. 8C) or conditional null lateral cortex (Fig. 8D; Tukey tests, p<0.05). Cultures containing lateral cortex from conditional null mutants produced average values for growth that were greater than those in cultures containing either control lateral cortex or no cortex, but the differences were not significant (Fig. 8D). Taking all these data together, the explant culture experiments showed that: (1) neither null nor control cortex attract thalamocortical axons; (2) neither null nor control cortex repel thalamocortical axons; (3) both null and control cortical angle region (and possibly null lateral cortex) stimulate general axon extension to a similar extent without providing direction specific outgrowth. Crucially, there is no evidence from these experiments that the angle or cortical regions of conditional null mutants either fail to stimulate thalamic axonal growth or generate signals that inhibit or repel thalamic axonal growth.

### Replacing null mutant cortex with control cortex allows thalamic axons to cross the PSPB

**Figure 9:**
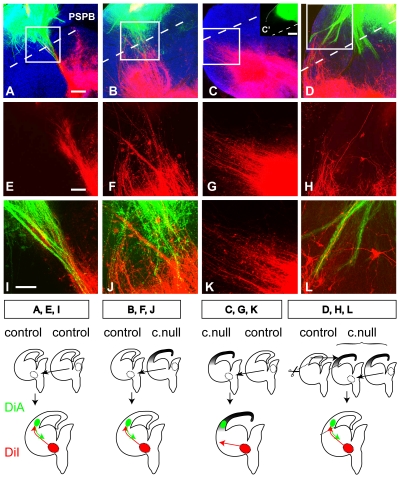
Replacing null mutant cortex with control cortex allows thalamic axons to cross the PSPB. (**A,B**) Thalamus from either control or conditional null embryos transplanted to the diencephalic-telencephalic border of control slices generated axons (DiI labelled, red) that crossed the PSPB in close association with descending cortical axons (DiA labelled, green). (**C**) When thalamus was transplanted to the diencephalic-telencephalic border of slices from conditional null embryos, thalamic axons traversed the subpallium but did not cross the PSPB. Injections of tracer into the cortex of these co-cultures revealed that, as expected from *in vivo* work, the conditional null cortex produced no descending axons (inset **C′**). (**D**) When thalamus was transplanted to the diencephalic-telencephalic border of slices from conditional null embryos whose cortex was replaced by control cortex, thalamic axons crossed the PSPB in close association with descending cortical axons provided from the control cortex. (**E–H**) Thalamic axons only are shown from the boxed areas in **A–D**. (**I–L**) Higher magnification images from the boxed areas in **A–D**. Nuclei were counterstained with TO-PRO-3 iodide. The diagrams underneath each column of photographs summarize the experimental paradigms and their outcomes. Scale bars: **A–D**, 50 µm; **E–L**, 25 µm.

The results in the previous section indicate that the failure of thalamic axons to cross the PSPB in conditional null mutants can not be explained by repulsive factors diffusing from the null mutant cortex, and that the growth promoting effects of the cortex is not diminished in conditional null mutants. This suggests that the loss of descending corticofugal axons in conditional null mutants might be the critical event preventing thalamic axons from crossing the PSPB. To test this further, we attempted to rescue the failure of thalamic axons from conditional null mutants to cross the PSPB by replacing null mutant cortex with control cortex capable of generating corticofugal axons. This was done in cultured E14.5 slices and the results are shown in Figure 9.

The first experiments were designed to confirm that the thalamus of conditional null embryos generates axons and that the subpallium of conditional null embryos allows these axons to traverse it in our culture system, as occurs *in vivo*. To test thalamic explants from either control or conditional null embryos, we transplanted them to the diencephalic-telencephalic border of control slices, from where they reliably generated axons that crossed the PSPB in close association with descending cortical axons (Fig. 9A,B,E,F,I,J; thalamic axonal crossing of the PSPB occurred in 7 out of 9 control and in 5 out of 6 conditional mutant thalamic transplants). When thalamus was transplanted to telencephalic slices from conditional null embryos (in which descending corticofugal axons were not observed, Fig. 9C'), thalamic axons traversed the subpallium but did not cross the PSPB in any case (Fig. 9C,G,K; n = 9 transplants), mimicking the failure observed *in vivo* in conditional null mutants.

We then tested the prediction that this failure of thalamic axons to cross the PSPB in conditional null telencephalon would be corrected by replacing the mutant cortex with control cortex capable of generating corticofugal axons. In 4 out of 5 transplants of control cortex into conditional null telencephalon, the control cortex produced axons that crossed into the subpallium. In all 4 cases, thalamic axons growing from and through conditional null thalamus and subpallium turned into the cortex in close association with the descending cortical axons (Fig. 9D,H,L). This result indicates that thalamic axons from conditional null mutants have the potential to cross the PSPB (Fig. 9B,F,J) and that this potential can be realised provided that the cortex supplies the necessary guidance (Fig. 9D,H,L).

## Discussion

The formation of axonal tracts in developing nervous systems is regulated by multiple mechanisms that use cues such as extracellular molecules, guidepost cells, and axon-to-axon interactions to guide growing axons to their specific target regions. Much of the evidence that such cues can play critical roles comes from work on non-mammalian species or on relatively anatomically simpler parts of mammalian nervous systems. Evidence is harder to obtain in the mammalian brain with its very large numbers of criss-crossing axonal pathways. This is exemplified very well in the development of the thalamocortical tract. Whereas there is now good evidence that extracellular guidance molecules such as slits, netrins, eph/ephrins, semaphorins and neuregulin-1 can help guide thalamic axons as they traverse ventral tissues en route towards the cortex [6], [7], [8], [10], [11], [35], [36], [37], and that cells in the subpallium provide a permissive corridor guiding thalamic axons through this region [10], evidence that axon-to-axon interactions are important has proved harder to obtain. It has been hypothesised, based on the spatial and temporal proximity of their development, that the descending axons of cortical neurons can guide the ascending thalamocortical axons [2], [4], [14], [16]. This hypothesis has been used as a possible explanation for the observation that abnormalities of both corticofugal and thalamocortical pathways are associated in some mutant mice, for example in *Pax6* and in *Celsr3* mutants [6], [7], [38], [39].

We used a strategy that prevented the formation of corticofugal axons while causing minimal disruption elsewhere, including the thalamus, subpallium, PSPB and adjacent ventral pallium. We used an *Emx1^Cre^* allele that does not cause recombination in the thalamus or subpallium to delete *Apc* in the cerebral cortex from about E9.5 on. APC is a multifunctional protein known to be upregulated in cortical precursors as they exit the cell cycle and migrate to the cortical plate to differentiate [40]. It is involved in regulating a variety of cellular processes including cell migration and axonogenesis [19], [21]. At ages leading up to the time around E13.5 when cortical efferents start to form and grow across the PSPB, *Emx1^Cre^*-induced deletion of *Apc* did not block completely the development of the cortex and this avoided the development of the major anatomical distortions that would have occurred in the rest of the brain if this tissue was entirely absent. The deletion caused a rather selective elimination of the production of postmitotic cortical neurons and hence a failure of the cortex to generate corticofugal axons across the PSPB.

The absence of these corticofugal axons was a likely explanation for the failure of thalamocortical axons to cross the PSPB, and this was strengthened by a variety of other experiments described in this paper whose results argued against other possible explanations. Since in conditional mutants the thalamocortical axons stop about 200 µm short of the deleted tissue, the deletion must be acting on the growth of thalamocortical axons at a distance. We found no evidence that the null mutant cortex was producing repulsive or growth-inhibitory molecules that might diffuse to confront the advancing thalamocortical axons. The growth promoting action of the cortex on thalamic axons was at least as strong in the null mutants as in the controls and cortical expression of Ig-Nrg1, which is believed to be the major factor promoting thalamic axonal growth in the vicinity of the PSPB [10], was similar in both.

These results imply that Ig-Nrg1 is not sufficient for attracting thalamic axons into the cortex. In agreement with previous studies, we found evidence that the cortex, in particular the angle region around which thalamic axons normally grow after crossing the PSPB, exerts a potent long-range growth-promoting but not a chemoattractive effect on thalamic axons [10], [13]. This raises the interesting possibility that the growth of thalamic axons approaching the PSPB is enhanced by molecules released from the cortex but guidance into the cortex is provided by descending cortical axons. Normally, the two mechanisms would combine to allow thalamic axons to cross the otherwise hostile environment of the PSPB. In the absence of descending axons, thalamic axons would continue to grow but would be deflected away from the PSPB, as observed in the conditional null mutants.

Our evidence indicates that the PSPB itself would not have been affected directly by the mutation in *Apc*, since cre recombinase was not active as far ventrally as the PSPB at the ages examined here. The PSPB showed the same morphological and molecular features in conditional null mutants and controls, suggesting that failure of thalamic axons to cross this region was not due to its being defective in some way. Indeed, we were able to induce thalamic axons across the PSPB of conditional null mutants by replacing the mutant cortex with control cortex, thereby re-establishing the corticofugal projections. This experiment confirmed that the thalamus, the subpallium and the PSPB from conditional null mutant embryos are able to produce, and to allow penetration by, thalamic axons capable of reaching the cortex.

It appears from these findings that the PSPB is not normally conducive to thalamic axonal penetration. It might offer a mechanical and/or a chemical impediment. It develops a striking radial glial fascicle that runs across the trajectory of thalamocortical axons [41], [42], [43] and it has a high density of cells, including those of the lateral cortical stream (LCS) that migrate across the path of the thalamocortical axons from the ventricular progenitors at the PSPB towards the ventral part of the subpallium [41]. To pass through the PSPB, thalamic afferents may need guidance from the environment. The timing of cortical efferent development suggests that theses axons could be an important guidance factor for thalamic axons to cross the PSPB. A small number of cortical axons descend from the cerebral cortex and start to cross the PSPB into the ventral telencephalon at around E12, an age when cells from the PSPB start to migrate along the putative route of the LCS. It is conceivable that the early cortical efferents that have passed the PSPB could provide a potential scaffold for the later born cortical axons as well as thalamic axons to cross the PSPB. The ventrally-directed LCS cells may generate a physical force that causes cortical efferents and thalamic afferents to bend in a dorsal to ventral direction and make a unique comb-shape curve along the LCS route. It is conceivable that in the absence of guidance across this LCS, thalamic axons turn and follow it ventrally. The role of descending cortical axons in drawing thalamic axons dorsally into the cortex might have evolved as a relatively simple mechanism allowing thalamic axons to avoid potentially distracting influences from other developing systems that are likely to be phylogenetically older than the neocortex. For future work it would be very interesting to conduct experiments to test the effects of removing the mechanical/chemical features of the PSPB on the growth of thalamocortical axons with and without cortical axons.

It has been demonstrated that cortical and thalamic axons pause in the vicinity of the PSPB for some hours before progressing toward their targets *in vivo* [4], [14]. It is conceivable that both cortical and thalamic axons are waiting to receive guidance factors to allow them to overcome the non-permissive features of the PSPB. Our results indicate that one essential guidance factor required by thalamic axons is an intimate interaction with descending cortical axons, which are likely to be among the necessary guidance factors that thalamic axons are waiting for to cross the PSPB. Our findings imply that, in normal development, at least some descending cortical axons might cross the PSPB ahead of thalamic axons so as to provide the necessary guidance and prevent abnormal thalamic axonal deflection away from the PSPB, such as that observed here in conditional mutants lacking any descending cortical efferents.

In summary, our study provides the most compelling evidence to date that descending cortical efferents are required to guide ascending thalamocortical axons across the PSPB. They indicate that in the absence of descending axons the PSPB is non-permissive to penetration by ascending thalamic axons. They add further support to the hypothesis that the navigation of thalamic axons from the subpallium into the cerebral cortex depends on two mechanisms: diffusible factors from the cortex stimulate the growth of thalamic axons while descending cortical efferents provide guidance dorsally across the PSPB.
